# Stigmasterol-Based Novel Low Molecular Weight/Mass Organic Gelators

**DOI:** 10.3390/molecules16119357

**Published:** 2011-11-08

**Authors:** Jana Šusteková, Pavel Drašar, David Šaman, Zdeněk Wimmer

**Affiliations:** 1 Department of Chemistry of Natural Compounds, Institute of Chemical Technology, Faculty of Food and Biochemical Technology, Technická 5, 166 28 Prague 6, Czech Republic; Email: Jana.Sustekova@gmail.com (J.Š.); Pavel.Drasar@vscht.cz (P.D.); 2 Isotope Laboratory, Institute of Experimental Botany AS CR, v.v.i., Vídeňská 1083, 142 20 Prague 4, Czech Republic; 3 Institute of Organic Chemistry and Biochemistry AS CR, v.v.i., Flemingovo nám. 2, 166 10 Prague 6, Czech Republic; Email: nmrsaman@gmail.com (D.Š.)

**Keywords:** stigmasterol, L-phenylalanine, ester, amide, supramolecule, low molecular weight/mass organic gelators

## Abstract

Conjugates consisting of stigmasterol and L-phenylalanine, interconnected through short-chained dicarboxylic acyls by ester and amide bonds, respectively, were synthesized as potential low molecular weight/mass organic gelators (LMWGs/LMMGs). Their physico-chemical properties were subjected to investigation, especially their ability to form gels reversibly based on changes of the environmental conditions. Other self-assembly properties detectable by UV-VIS traces were measured in systems consisting of two miscible solvents (water/acetonitrile) with varying solvent ratios and using constant concentrations of the studied compounds. Partition and diffusion coefficients and solubility in water were calculated for the target conjugates. The conjugate **3a** was the only compound from this series capable of forming a gel in 1-octanol. All three conjugates **3a**–**3c** displayed supramolecular characteristics in the UV-VIS spectra.

## 1. Introduction

Investigations in the area of supramolecular chemistry during the last two decades have resulted in the discovery of a large number of new compounds displaying self-assembly characteristics, often assisted by solvents. Many of those novel compounds have been proved to form reversible gels, and since that time, as Professor Dastidar has stated [1]: “Gels are everywhere!”, and the number of papers dealing with different aspects of this field of supramolecular chemistry has been steadily increasing. The most frequent components of self-assembly systems are heterocycles and other aromatic systems and amino acids (both aromatic and non-aromatic in nature), combined with steroid molecules, mono- and oligosaccharides, fatty acyls and even hydrocarbons and organometallic compounds [1,2,3]. Steroid compounds, particularly the bile acids [4,5,6,7,8], but also other types of steroids, like phytosterols and phytoecdysteroids [2] can be a basis for designing supramolecules in combination with other small molecules, e.g., amino acids, nucleotides, heterocycles, *etc.* [1,2,3]. A specific group among such structures is represented by steroid-based anion and cation receptors and transporters. Ion transportation across biological membranes represents a fundamental process essential for homeostasis in multicellular life forms. Biological systems use a number of mechanisms by which hydrophilic ions pass through phospholipid bilayers possessing hydrophobic nature. Steroid compounds represent convenient natural molecules capable of assisting in transportation of ions through the hydrophobic bilayers or modifying the gelator properties [9]. In turn, several types of steroid derivatives with other small molecules of natural or synthetic origin have already been proved to become a specific part of supramolecular gelators, *i.e.*, low molecular weight/mass organic gelators (LMWGs/LMMGs). Recently, we have published a paper dealing with the synthesis of phytosterol-amino acid conjugates and investigation of their physico-chemical characteristics as supramolecular gelators [2]. Among steroid molecules, phytosterols have not yet received adequate attention, despite the fact that these natural products can be relatively easily extracted from numerous plants. Phytosterols are biogenetic precursors of phytoecdysteroids, which are important plant products acting mainly in plant defense systems. Phytosterols are structurally close to cholesterol, and many of them display important pharmacological activities, including cholesterol lowering activity in the human body.

Amino acids represent key products for humans and other living organisms. Enantiomerically pure non-natural amino acids are often used as valuable intermediates in the preparation of pharmaceuticals [10,11]. In the other hand, both enantiomerically pure natural and non-natural amino acids may become parts of molecules of low molecular weight organic gelators (LMWGs) [2]. Amino acids bear a charge on their molecules. In combination with steroid compounds like phytosterols, the resulting conjugates may provide H-bond donor functionality combined with the lipophilic nature of phytosterol molecules [2]. Hydrogen bonds, π-π stacking of aromatic rings and multiple bond systems represent the key forces responsible for supramolecular self-assembly of the studied systems.

The objectives of this investigation were: (a) a synthesis of conjugates of stigmasterol and L-phenylalanine, connected by ester and amide bonds through short-chained dicarboxylic acyl compounds; and (b) a study of the physico-chemical properties of these novel potential LMWGs.

## 2. Results and Discussion

**Scheme 1:**
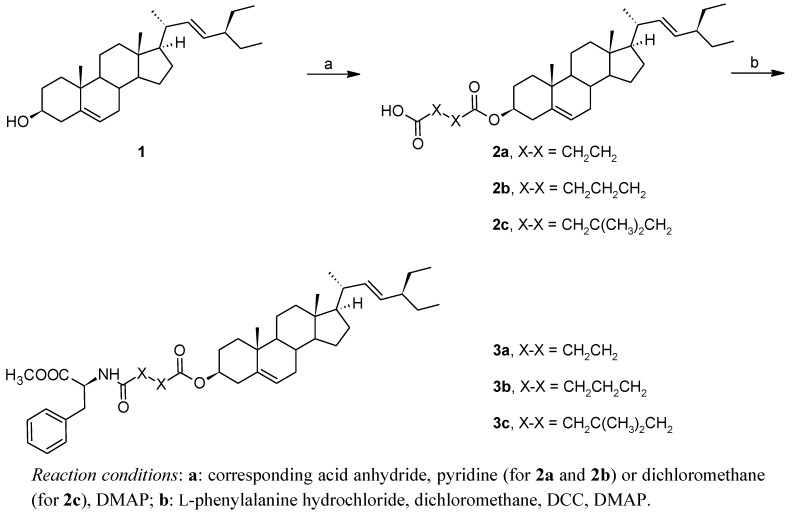
Synthetic procedure.

Hemiesters of stigmasterol **2a**–**2c** were prepared by reacting the sterol **1** with the corresponding acid anhydride in either dry pyridine (in the case **2a** and **2b**) or in dry dichloromethane (for **2c**) in the presence of 4-dimethylaminopyridine (DMAP). Using pyridine in the synthesis of **2c** resulted in only a 13% yield. The subsequent amide bond formation between the amino group of methyl L-phenylalanine and the corresponding hemiesters **2a**–**2c** was carried out in dichloromethane in the presence of dicyclohexylcarbodiimide (DCC) as an auxiliary condensation reagent and small quantity of DMAP as reaction promoter, and resulted in the target conjugates **3a**–**3c** (Scheme 1).

**Figure 1:**
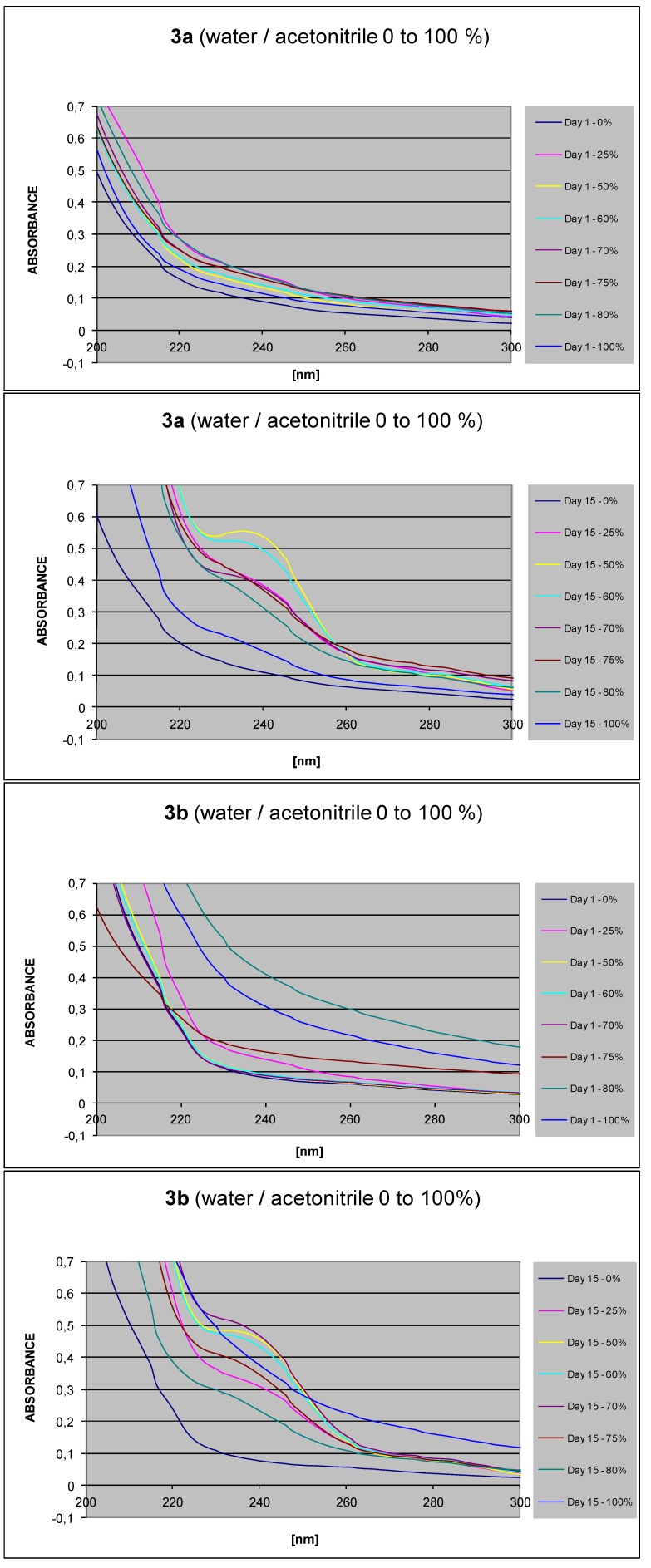
UV-VIS traces measured for **3a** and **3b** in a water/acetonitrile system with varying ratios of both solvents (0 to 100%) using a constant concentration of **3a** and **3b** (data taken on Day 1 and the Day 15 of the measurement).

The obtained conjugates **3a**–**3c** were subjected to a screening of their supramolecular properties. Their UV-VIS spectra were recorded in solvent systems consisting of water and an organic solvent miscible with water (acetonitrile in our experiments). The concentration of the studied compound was constant in all solutions, and the ratio of both solvents was changed stepwise in 5–25% steps. Under the described conditions, the UV-VIS spectra of the compounds able to show self-assembly properties, display irregularities indicating formation of supramolecular structures in the studied systems consisting of two solvents. Figure 1 shows several examples of those results and irregularities. When the compound forms no supramolecular structures in the solutions in two-solvent-based systems, then the absorbance intensity follows the sequence of solvent ratio change. However, when the compound is able to form supramolecular structures, then the absorbance intensity is responsive to structural changes due to hydrogen bond formation (supramolecular polymer) or due to the π-π stacking of aromatic rings. Formation of both types of van der Waals forces may be accompanied by absorbance maximum shift in different solvent ratio solutions. Supramolecular structures are formed with different rates, and the structure of supramolecular systems can change during the longer time, as shown in Figure 1. It follows from this figure that the conjugates **3a** and **3b** display supramolecular characteristics in their UV-VIS spectra. The supplementary material contains the whole sequence of long-term UV-VIS measurement traces, supporting this finding.

**Figure 2:**
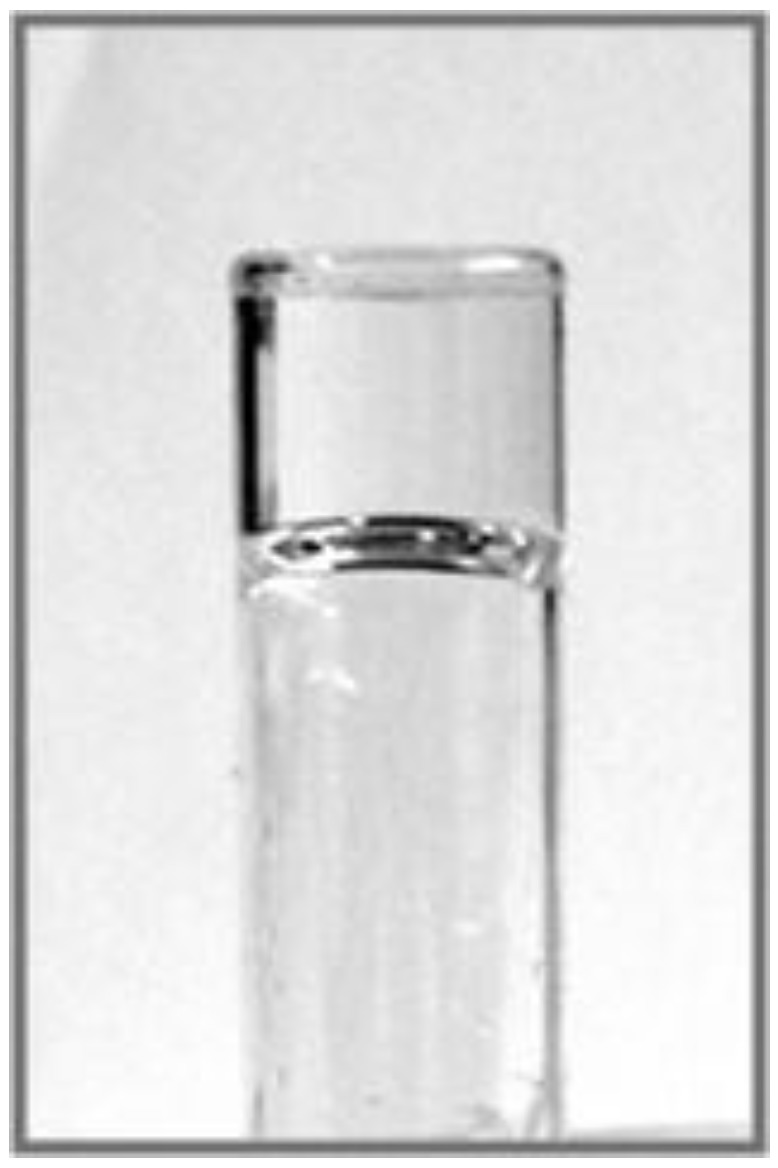
Gel formed by **3a** in 1-octanol.

Based on the UV-VIS measurement results, several solvents were tested to determine if the studied compounds would display the ability to form gels. Thus, benzene, toluene, dichloromethane, methanol and 1-octanol were tested as solvents in gelation ability screening experiments for the new compounds. Table 1 shows the results of this screening. Only compound **3a** formed a reversible gel in 1-octanol (Figure 2), while the compounds **3b** and **3c** formed solutions in this solvent. All tested compounds formed precipitates in methanol, and solutions in all other tested solvents. Compound **3a** formed the gel in 1-octanol upon cooling in a refrigerator. The gel was transformed into a solution by slowly increasing the temperature to the laboratory temperature, and the gel reversibly formed by repeated cooling in the refrigerator.

**Table 1:** Ability of the conjugates **3a**–**3c** to form gels.

Compound	Solvent				
	Benzene	Toluene	Dichloromethane	Methanol	1-Octanol
**3a**	solution	solution	solution	precipitation	**gel**
**3b**	solution	solution	solution	precipitation	solution
**3c**	solution	solution	solution	precipitation	solution

## 3. Experimental

### 3.1. General

The ^1^H-NMR and the ^13^C-NMR spectra were recorded on a Bruker AVANCE 600 MHz spectrometer at 600.13 MHz and 150.90 MHz in deuteriochloroform using tetramethylsilane (δ = 0.0) as internal reference. ^1^H-NMR data are presented in the following order: chemical shift (δ) expressed in ppm, multiplicity (s, singlet; d, doublet; t, triplet; q, quartet; m, multiplet), coupling constants in Hertz, number of protons. Infrared spectra were measured with a Nicolet 205 FT-IR spectrometer. Mass spectra were measured with a Waters ZMD mass spectrometer in a positive ESI mode. TLC was carried out on silica gel plates (Merck 60F254) and the visualization was performed both, by UV detection and by spraying with the methanolic solution of phosphomolybdic acid (5%) followed by heating. Elemental analyses were performed on a Perkin Elmer 2400, series II CHNS/O analyzer (USA). Melting points were determined on a Kofler MHK melting point apparatus (Franz Küstner Nacht, KG, Dresden, Germany) and are uncorrected. All chemicals and solvents were purchased from regular commercial sources in analytical grade and the solvents were purified by general methods before use. For column chromatography, silica gel 60 (0.063–0.200 mm) from Merck was used. ACD/Labs software, ACD/logD DB, version 12.01, was used for calculation of solubility, partition coefficient (log P) and distribution coefficient (log D) of the prepared compounds.

### 3.2. 4-Oxo-4-[(3β,22E)-stigmasta-5,22-dien-3-yloxy]butanoic Acid (**2a**), 5-Oxo-5-[(3β,22E)-stigmasta-5,22-dien-3-yloxy]pentanoic Acid (**2b**) and 5-Oxo-5-[(3β,22E)-stigmasta-5,22-dien-3-yloxy]-3,3-dimethylpentanoic Acid (**2c**)

A catalytic amount of 4-dimethylaminopyridine (DMAP; 0.2 g, 1.637 mmol) was added to a solution of stigmasterol (**1**, 4.0 g, 9.693 mmol) and the anhydride of the corresponding dicarboxylic acid (15.188 mmol) in either dry pyridine (17.5 mL; in the preparation of **2a** and **2b**) or dry dichloromethane (10 mL; in the preparation of **2c**); path **a** in Scheme 1. The mixture was stirred at room temperature for 7 days and then poured onto a mixture of ice (30 mL)/hydrochloric acid (15 mL). The organic layer was extracted with chloroform (5 × 40 mL), the combined extracts were dried over sodium sulfate, and the solvent was finally evaporated to dryness. The residue was purified by column chromatography using a chloroform/methanol gradient (100:0 to 60:1) mixture as mobile phase. Yields: **2a**: 4.62 g (93%), **2b**: 3.41 g (67%), **2c**: 3.07 g (57%).

*4-Oxo-4-[(3β,22E)-stigmasta-5,22-dien-3-yloxy]butanoic acid* **(2a**): ^1^H-NMR: 5.37 (ddt, 1H, *J *= 1.3; 1.3; 1.8; 5.0 Hz, H-6´), 5.15 (dd, 1H, *J *= 8.7; 15.2 Hz, H-22´), 5.01 (dd, 1H, *J *= 8.9; 15.2Hz, H-23´), 4.63 (dddd, 1H, *J *= 4.2; 7.1; 9.5; 11.6 Hz, H-3´), 2.61 (m, 2H, H-31´), 2.68 (m, 2H, H-32´), 2.30–2.33 (m, 2H, H-4´), 2.00–2.06 (m, 1H, H-20´), 1.55–1.60 (m) + 1.80–1.86 (m, 2H, H-2´), 1.52–1.56 (m, 1H, H-24´), 1.51–1.55 (m, 1H, H-25´), 1.46–1.51 (m, 2H, H-11´), 1.46–1.51 (m, 1H, H-8´), 1.37–1.41 (m) + 1.93–2.01 (m, 2H, H-7´), 1.14–1.19 (m) + 1.41–1.46 (m, 2H, H-28´), 1.21–1.28 (m) + 1.70 (ddd, 2H, *J* = 6.1; 9.8; 13.0 Hz, H-16´), 1.12–1.17 (m, 1H, H-17´), 1.09–1.12 (m) + 1.83–1.88 (m, 2H, H-1´), 1.07 (ddd, 1H, *J *= 6.2; 11.3; 12.6 Hz, H-14´), 1.06–1.11 (m) + 1.96-2.00 (m, 2H, H-12´), 1.021 (d, 3H, *J* = 6.6 Hz, H-21´), 1.020 (s, 3H, H-19´), 1.01–1.06 (m) + 1.50–1.56 (m, 2H, H-15´), 0.95 (ddd, *J* = 5.1; 10.9; 12.1 Hz, H-9´), 0.846 (d, 3H, *J* = 6.5 Hz, H-26´), 0.805 (t, 3H, *J *= 7.4 Hz, H-29´), 0.795 (d, 3H, *J* = 6.6 Hz, H-27´), 0.695 (s, 3H, H-18´). ^13^C-NMR: 177.54 (s, C-33´), 171.53 (s, C-30´), 139.49 (s, C 5´), 138.31 (d, C-22´), 129.24 (d, C-23´), 122.72 (d, C-6´), 74.53 (d, C-3´), 56.75 (d, C 14´), 55.88 (d, C-17´), 51.21 (d, C-24´), 49.98 (d, C-9´), 42.17 (s, C-13´), 40.51 (d, C-20´), 39.59 (t, C 12´), 37.98 (t, C-4´), 36.56 (s, C-10´), 36.92 (t, C-1´), 31.87 (d, C-25´), 31.87 (t, C-8´), 31.81 (t, C-7´), 29.20 (t, C-31´), 28.91 (t, C-32´), 28.89 (t, C-16´), 27.66 (t, C-2´), 25.40 (t, C-28´), 24.34 (t, C-15´), 21.22 (q, C-21´), 21.09 (q, C-26´), 20.99 (t, C-11´), 19.30 (q, C-19´), 18.97 (q, C-27´), 12.25 (q, C-29´), 12.03 (q, C-18´). IR (KBr): [cm^−1^] 1732 vs and 1715 s (C=O), 1381 w (CH_3_), 1178 s (C-O). For C_33_H_52_O_4_ (512.75) calcd.: 77.29% C, 10.22% H; found: 77.15% C, 10.41% H. MS (ESI, 20 eV): [M+Na]^+^ 535.

*5-Oxo-5-[(3β,22E)-stigmasta-5,22-dien-3-yloxy]pentanoic acid *(**2b**): ^1^H-NMR: 5.37 (ddt, 1H, *J* = 1.2; 1.2; 2.0, 5.8 Hz, H-6´), 5.15 (dd, 1H, *J* = 8.7; 15.2 Hz, H-22´), 5.02 (ddd, 1H, *J* = 0.6; 9.0; 15.2 Hz, H-23´), 4.62 (dddd, 1H, *J* = 4.3; 7.5; 9.4; 11.3 Hz, H-3´), 2.44 (t, 2H, *J* = 7.3 Hz, H-31´), 2.38 (t, 2H, *J* = 7.3 Hz, H-33´), 2.39–2.33 (m, 2H, H-4´), 2.00–2.07 (m, 1H, H-20´), 1.96 (m, 2H, H-32´), 1.55–1.62 (m) + 1.83–1.86 (m, 2H, H-2´), 1.53–1.57 (m, 1H, H-24´), 1.51–1.54 (m, 1H, H-25´), 1.48–1.53 (m, 2H, H-11´), 1.48–1.53 (m, 1H, H-8´), 1.39–1.44 (m) + 1.94–2.00 (m, 2H, H-7´), 1.14–1.19 (m) + 1.41–1.45 (m, 2H, H-28´), 1.22–1.28 (m) + 1.70 (ddd, 2H, *J* = 6.1; 9.8; 13.0 Hz, H-16´), 1.12–1.16 (m, 1H, H-17´), 1.09–1.12 (m) + 1.83–1.88 (m, 2H, H-1´), 1.07 (ddd, 1H, *J* = 6.1; 11.3; 12.6 Hz, H-14´), 1.08–1.13 (m) + 1.97–2.01 (m, 2H, H-12´), 1.022 (d, 3H, *J* = 6.6 Hz, H-21´), 1.020 (s, 3H, H-19´), 1.01–1.05 (m) + 1.51–1.57 (m, 2H, H-15´), 0.96 (ddd, *J* = 5.0; 10.9; 12.2 Hz, H-9´), 0.846 (d, 3H, *J* = 6.5 Hz, H-26´), 0.799 (t, 3H, *J* = 7,4 Hz, H-29´), 0.796 (d, 3H, *J* = 6.7 Hz, H-27´), 0.696 (s, 3H, H-18´). ^13^C-NMR: 178.06 (s, C-34´), 172.29 (s, C-29´), 139.55 (s, C 5´), 138.31 (d, C-22´), 129.23 (d, C-23´), 122.68 (d, C-6´), 74.09 (d, C-3´), 56.75 (d, C 14´), 55.88 (d, C-17´), 51.21 (d, C-24´), 49.99 (d, C-9´), 42.17 (s, C-13´), 40.51 (d, C-20´), 39.59 (t, C 12´), 38.09 (t, C-4´), 36.94 (t, C-1´), 36.57 (s, C-10´), 33.50 (t, C-31´), 32.83 (t, C 33´), 31.87 (d, C-25´), 31.87 (t, C-8´), 31.82 (t, C-7´), 28.91 (t, C-16´), 27.76 (t, C-2´), 25.41 (t, C-28´), 24.34 (t, C-15´), 21.21 (q, C-21´), 21.09 (q, C-26´), 20.99 (t, C-11´), 19.88 (t, C-32´), 19.30 (q, C-19´), 18.96 (q, C-27´), 12.25 (q, C-29´), 12.03 (q, C-18´). IR (KBr): [cm^−1^] 1739 vs and 1712 s (C=O), 1380 w (CH_3_), 1180 s (C-O). For C_34_H_54_O_4_ (526.77) calcd.: 77.52% C, 10.33% H, found: 77.34% C, 10.31% H. MS (ESI, 20 eV): [M+Na]^+^ 549.

*5-Oxo-5-[(3β,22E)-stigmasta-5,22-dien-3-yloxy]-3,3-dimethylpentanoic* *acid* (**2c**): ^1^H-NMR: 5.38 (ddt, 1H, *J* = 1.3; 1.3; 2.0; 5.0 Hz, H-6´), 5.15 (dd, 1H, *J* = 8.6; 15.1 Hz, H-22´), 5.02 (dd, 1H, *J* = 8.9; 15.1 Hz, H-23´), 4.65 (dddd, 1H, *J* = 4.1; 7.7; 9.0; 11.3 Hz, H-3´), 2.46 (s, 2H, H-33´), 2.41 (s, 2H, H-31´), 2.31–2.33 (m, 2H, H-4´), 2.02–2.08 (m, 1H, H-20´), 1.57–1.62 (m) + 1.84–1.89 (m, 2H, H-2´), 1.52–1.56 (m, 1H, H-25´), 1.52–1.56 (m, 1H, H-24´), 1.49–1.55 (m, 1H, H-8´), 1.47–1.53 (m, 2H, H-11´), 1.43–1.49 (m) + 1.95–2.00 (m, 2H, H-7´), 1.23–1.28 (m) + 1.68–1.73 (m, 2H, H-16´), 1.17–1.21 (m) + 1.97–2.02 (m, 2H, H-12´), 1.14–1.18 (m) + 1.41–1.44 (m, 2H, H-28´), 1.14 (s, 3H, H-35´), 1.13–1.18 (m, 1H, H-17´), 1.12–1.16 (m) + 1.84–1.89 (m, 2H, H-1´), 1.03–1.07 (m) + 1.53–1.57 (m, 2H, H-15´), 1.02 (d, 3H, *J* = 6.6 Hz, H-21´), 1.02 (s, 1H, H-19´), 0.99–1.05 (m, 1H, H-14´), 0.94–0.99 (m, 1H, H-9´), 0.85 (d, 3H, *J* = 6.5 Hz, H-26´), 0.81 (t, 3H, *J* = 7.3 Hz, H-29´), 0.80 (d, 3H, *J* = 6.5 Hz, H-27´), 0.70 (s, 3H, H-18´). ^13^C-NMR: 175.57 (s, C-34´), 172.17 (s, C-30´), 139.44 (s, C 5´), 138.30 (d, C-22´), 129.28 (d, C-23´), 122.80 (d, C-6´), 74.38 (d, C-3´), 56.76 (d, C 14´), 55.92 (d, C-17´), 51.22 (d, C-24´), 50.00 (d, C-9´), 45.42 (t, C-31´), 45.21 (t, C-33´), 42.19 (s, C-13´), 40.50 (d, C-20´), 39.30 (t, C 12´), 38.11 (t, C-4´), 36.95 (t, C-1´), 36.60 (s, C-10´), 33.50 (t, C-31´), 32.83 (t, C-33´), 31.87 (d, C-25´), 31.87 (t, C-8´), 31.83 (t, C-7´), 28.90 (t, C-16´), 27.98 (q, C-35´), 27.81 (t, C-2´), 25.40 (t, C-28´), 21.22 (q, C-21´), 21.08 (q, C-26´), 21.00 (t, C-11´), 19.31 (q, C-19´), 18.97 (q, C-27´), 12.24 (q, C-29´), 12.04 (q, C-18´). IR (KBr): [cm^−1^] 1733 vs and 1714 s (C=O), 1390 w (CH_3_), 1178 s (C-O). For C_36_H_58_O_4_ (554.82) calcd.: 77.93% C, 10.54% H; found: 77.77% C, 10.51% H. MS (ESI, 20 eV): [M+Na]^+^ 577.

### 3.3. Methyl N-{4-oxo-4-[(3β,22E)-stigmasta-5,22-dien-3-yloxy]butanoyl}-L-phenylalanine (**3a**), Methyl N-{5-oxo-5-[(3β,22E)-stigmasta-5,22-dien-3-yloxy]pentanoyl}-L-phenylalanine (**3b**) and Methyl N-{5-oxo-5-[(3β,22E)-stigmasta-5,22-dien-3-yloxy]-3,3-dimethylpentanoyl}-L-phenylalanine (**3c**)

A solution of stigmasteryl hemiester (**2a**–**2c**, 0.195 mmol), L-phenylalanine hydrochloride (0.195 mmol), DCC (0.234 mmol) and DMAP (0.058 mmol) in dry dichlomethane (10 mL), containing several drops of dry pyridine (to neutralize the hydrogen chloride formed), was stirred at room temperature for 2 days; path **b** in Scheme 1. The solvent was then evaporated, and the residue was purified by column chromatography on silica gel, using a chloroform/methanol gradient (100:0 to 80:1) mixture as mobile phase. Yields: **3a**: 97%, **3b**: 96%, **3c**: 89%.

*Methyl N-{4-oxo-4-[(3β,22E)-stigmasta-5,22-dien-3-yloxy]butanoyl}-L-phenylalanine *(**3a**): ^1^H-NMR: 7.27–7.31 (m, 1H, H-38´), 7.23–7.26 (m, 1H, H-39´), 7.09–7.12 (m, 1H, H-37´), 5.35 (m, 1H, H-6´), 5.15 (dd, 1H, *J* = 8.8; 15.2 Hz, H-22´), 5.02 (dd, 1H, *J* = 8.8; 15.2 Hz, H-23´), 4.88 (dt, 1H, *J* = 5.8; 5.8; 7.7 Hz, H-34´), 4.58–4.64 (m, 1H, H-3´), 3.72 (s, 3H, H-41´), 3.09 (dd, 2H, *J* = 5.6; 13.9 Hz, H-35´)+3.14 (dd, 2H, *J* = 5.8; 13.9 Hz, H 35´), 2.59 (ddd, 2H, *J* = 6.0; 7.0; 17.1 Hz, H-31´)+2.63 (dt, 1H, *J* = 7.2; 7.2; 17.1 Hz, H-31´), 2.45–2.51 (m, 2H, H-4´), 2.28–2.32 (m, 2H, H-32´), 1.02 (d, 3H, *J* = 6.7 Hz, H-21´), 1.01 (s, 3H, H-19´), 0.85 (d, 3H, *J* = 6.5 Hz, H-26´), 0.80 (t, 3H, *J* = 7.4 Hz, H-29´), 0.79 (d, 3H, *J* = 6.5 Hz, H-27´), 0.69 (s, 3H, H-18´). ^13^C-NMR: 172.14 (s, C-33´), 171.92 (s, C-40´), 170.99 (s, C-30´), 139.58 (s, C-5´), 138.31 (d, C-22´), 135.78 (s, C-36´), 129.27 (d, C-37´), 129.23 (d, C-23´), 128.56 (t, C-38´), 127.11 (d, C-39´), 122.66 (d, C-6´), 74.37 (d, C-3´), 56.75 (d, C-14´), 55.88 (d, C-17´), 53.13 (d, C-34´), 52.32 (q, C-41´), 51.21 (d, C-24´), 50.00 (d, C-9´), 42.17 (s, C-13´), 40.50 (d, C-20´), 39.59 (t, C 12´), 38.02 (t, C-4´), 37.86 (t, C-35´), 36.94 (t, C-1´), 36.57 (s, C-10´), 31.87 (d, C-25´), 31.87 (t, C-8´), 31.81 (t, C-7´), 30.89 (t, C-32´), 29.66 (t, C-31´), 28.91 (t, C-16´), 27.68 (t, C-2´), 25.40 (t, C-28´), 24.33 (t, C-15´), 21.20 (q, C-21´), 21.09 (q, C-26´), 20.98 (t, C-11´), 19.30 (q, C-19´), 18.96 (q, C-27´), 12.25 (q, C-29´), 12.02 (q, C-18´). IR (KBr): [cm^−1^] 3282m (-CO-NH-), 2956 m and 2947 m (-CH_2_), 2890 m and 2869 m (-CH_3_), 1743 vs and 1644 s (C=O), 1216 s (Ar), 1177 s (C-O). For C_43_H_63_NO_5_ (673.97) calcd.: 76.74% C, 9.36% H, found: 76.64% C, 9.34% H. MS (ESI, 20 eV): [M+Na]^+^ 696. Calculated partition coefficient: neutral form logP = 11.22 ± 0.51, form charged on nitrogen logP = 9.22 ± 1.00. Calculated diffusion coefficient for neutral pH: logD = 11.22. Solubility in pure water at pH = 7: 2.9606 × 10^−7^ mg·mL^−1^.

*Methyl N-{5-oxo-5-[(3β,22E)-stigmasta-5,22-dien-3-yloxy]pentanoyl}-L-phenylalanine* (**3b**): ^1^H-NMR: 7.27–7.31 (m, 1H, H-39´), 7.23–7.26 (m, 1H, H-40´), 7.08–7.11 (m, 1H, H-38´), 5.36–5.38(m, 1H, H-6´), 5.15 (dd, 1H, *J* = 8.7; 15.1 Hz, H-22´), 5.02 (dd, 1H, *J* = 8.7; 15.1 Hz, H-23´), 4.90 (dt, 1H, *J* = 5.9; 5.9; 7.9 Hz, H-35´), 4.58–4.64 (m, 1H, H-3´), 3.73 (m, 3H, H-42´), 3.16 (dd, 2H, *J* = 5.8; 13.9 Hz, H-36´) + 3.08 (dd, 2H, *J* = 6.1; 13.9 Hz, H-36´), 2.29–2.31 (m, 2H, H-4´), 2.26–2.34 (m, 2H, H-31´), 2.20–2.27 (m, 2H, H-33´), 1.91 (m, 2H, H-32´), 1.02 (d, 3H, *J* = 6.6 Hz, H-21´), 1.02 (s, 3H, H-19´), 0.85 (d, 3H, *J* = 6.6 Hz, H-26´), 0.81 (t, 3H, *J* = 7.3 Hz, H-29´), 0.79 (d, 3H, *J* = 6.7 Hz, H-27´), 0.70 (s, 3H, H-18´). ^13^C-NMR: 172.51 (s, C-34´), 172.06 (s, C-41´), 171.72 (s, C-30´), 139.50 (s, C-5´), 138.31 (d, C-22´), 135.80 (s, C-37´), 129.23 (d, C-23´), 129.21 (d, C-38´), 128.59 (d, C-39´), 127.14 (d, C-40´), 122.67 (d, C-6´), 74.00 (d, C-3´), 56.75 (d, C-14´), 55.88 (d, C-17´), 52.97 (d, C-35´), 52.35 (q, C-42´), 51.21 (d, C-24´), 50.00 (d, C-9´), 42.17 (s, C-13´), 40.51 (d, C-20´), 40.00 (d, C-9´), 39.59 (t, C-12´), 38.10 (t, C-4´), 37.89 (t, C-36´), 36.95 (t, C-1´), 36.58 (s, C-10´), 35.25 (t, C-33´), 33.51 (t, C-31´), 31.87 (d, C-25´), 31.87 (t, C-8´), 31.82 (t, C-7´), 28.91 (t, C-16´), 27.78 (t, C-2´), 25.40 (t, C-28´), 24.33 (t, C-15´), 21.21 (q, C 21´), 21.10 (q, C-26´), 20.99 (t, C-11´), 20.78 (t, C-32´), 19.31 (q, C-19´), 18.96 (q, C-27´), 12.25 (q, C-29´), 12.03 (q, C-18´). IR (KBr): [cm^−1^] 3072 m (-CO-NH-), 2960 m and 2934 m (-CH_2_), 2892 m and 2880 m (-CH_3_), 1738 vs and 1653 s (C=O), 1218 s (Ar), 1181 s (C-O). For C_44_H_65_NO_5_(687.99) calcd.: 76.93% C, 9.46% H; found: 76.89% C, 9.46% H. MS (ESI, 20 eV): [M+Na]^+^ 710. Calculated partition coefficient: neutral form logP = 11.49 ± 0.50; form charged on nitrogen logP = 9.49 ± 1.00. Calculated diffusion coefficient for neutral pH: logD = 11.49. Solubility in pure water at pH = 7: 2.0952 × 10^−7^ mg·mL^−1^.

*Methyl N-{5-oxo-5-[(3β,22E)-stigmasta-5,22-dien-3-yloxy]-3,3-dimethylpentanoyl}-L-phenylalanine *(**3c**): ^1^H-NMR: 7.27–7.30 (m, 1H, H-42´), 7.22–7.25 (m, 1H, H-43´), 7.15–7.18 (m, 1H, H-41´), 5.40–5.42 (m, 3H, H-6´), 5.16 (dd, 1H, *J* = 8.7; 15.2 Hz, H-22´), 5.02 (dd, 1H, *J* = 8.6; 15.2 Hz, H-23´), 4.87 (dt, 1H, *J* = 5.4; 8.1; 8.1 Hz, H-38´), 4.60–4.66 (m, 1H, H-3´), 3.72 (s, 3H, H-44´), 3.19 (dd, 1H, *J* = 5.4; 14.1 Hz, H-39´), 3.00 (dd, 1H, *J* = 8.1; 14.1 Hz, H-39´), 2.39 (d, 1H, *J* = 12.5, H-31´), 2.30 (d, 1H, *J* = 12.7, H-33´), 2.18 (d, 1H, *J* = 12.7 Hz, H-33´), 2.05 (d, 2H, *J* = 12.5 Hz, H-31´), 1.03 (d, 3H, *J* = 6.6 Hz, H-21´), 1.03 (s, 3H, H-36´), 1.03 (s, 3H, H-19´), 0.99 (s, 3H, H-35´), 0.85 (d, 3H, *J* = 6.7 Hz, H-26´), 0.81 (t, 3H, *J* = 7.4 Hz, H-29´), 0.81 (d, 3H, *J* = 6.6 Hz, H-27´), 0.71 (s, 3H, H-18´). ^13^C-NMR: 172.67 (s, C-37´), 172.49 (s, C-34´), 171.102(s, C-30´), 139.46 (s, C-5´), 138.29 (d, C-22´), 136.28 (s, C-40´), 129.26 (d, C-23´), 129.12 (d, C-42´), 128.52 (d, C-41´), 126.97 (d, C-43´), 122.80 (d, C-6´), 74.25 (d, C-3´), 56.76 (d, C-14´), 55.89 (d, C-17´), 53.18 (d, C-38´), 52.21 (q, C-44´), 51.21 (d, C-24´), 50.02 (d, C-9´), 46.83 (t, C-33´), 44.62 (t, C-31´), 42.18 (s, C-13´), 40.51 (d, C-20´), 39.59 (t, C 12´), 38.16 (t, C-4´), 37.80 (t, C-39´), 36.96 (t, C-1´), 36.60 (s, C-10´), 33.65 (s, C-32´), 31.88 (d, C-25´), 31.88 (t, C-7´), 31.81 (d, C-8´), 28.91 (t, C-16´), 28.72 (q, C-36´), 28.68 (q, C-35´), 27.78 (t, C-2´), 25.40 (t, C-28´), 24.34 (t, C-15´), 21.21 (q, C-21´), 21.10 (q, C-26´), 20.99 (t, C-11´), 19.30 (q, C-19´), 18.96 (q, C-27´), 12.25 (q, C-29´), 12.03 (q, C-18´). IR (KBr): [cm^−1^] 3318 m (-CO-NH-), 2956 m and 2937 m (-CH_2_), 2890 m and 2869 s (-CH_3_), 1731 vs and 1653 s (C=O), 1220 s (Ar), 1177 s (C-O). For C_46_H_69_NO_5_ (716.05) calcd.: 77.27% C, 9.65% H; found: 77.23% C, 9.64% H. MS (ESI, 20 eV): [M+Na]^+^ 738. Calculated partition coefficient: neutral form logP = 12.26 ± 0.51, form charged on nitrogen logP = 10.26 ± 1.00. Calculated diffusion coefficient for neutral pH: logD = 12.26. Solubility in pure water at pH = 7: 9.4142 × 10^−8^ mg·mL^−1^.

## 4. Conclusions

Several novel stigmasterol conjugates were synthesized, and their solvent-assisted supramolecular abilities to self-assemble, and their ability to form gels were studied. The conjugates **3a** and **3b** showed irregularities in their UV-VIS spectra in the sequences of change of the solvent ratios, and supramolecular characteristics were proven with all conjugates **3a**, **3b** and **3c**. However, only the conjugate **3a** was able to form a gel reversibly. In turn, it is evident that the conjugates **3b** and **3c** are capable of forming organized, solvent-mediated supramolecular systems in solution. Formation of visible gels cannot usually be predicted, and is strongly dependent on the selection of the solvent.

## Supplementary materials

Supplementary File 1

Supplementary File 2
