# Supplementary material for: Stigmasterol-Based Novel Low Molecular Weight/Mass Organic Gelators
Source: Molecules. 2011 Nov 8;16(11):9357–67. doi: 10.3390/molecules16119357 (PMC6264699; doi:10.3390/molecules16119357)
Supplement: Supplementary File 1 [file molecules-16-09357-s001.pdf]

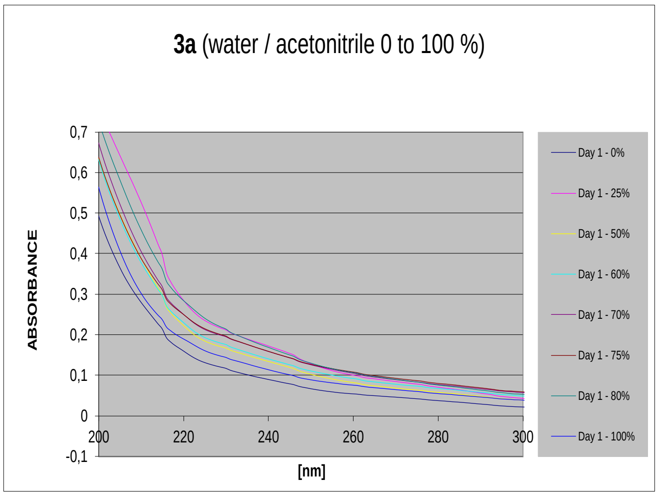
3a (water / acetonitrile 0 to 100 %)
ABSORBANCE
0,7
0,6
0,5
0,4
0,3
0,2
0,1
0
-0,1
200
220
240
260
280
300
[nm]
Day 1 - 0%
Day 1 - 25%
Day 1 - 50%
Day 1 - 60%
Day 1 - 70%
Day 1 - 75%
Day 1 - 80%
Day 1 - 100%

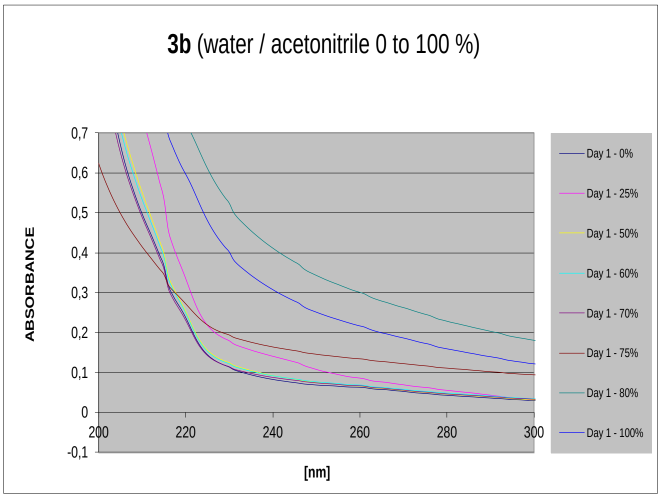
3b (water / acetonitrile 0 to 100 %)
ABSORBANCE
0,7
0,6
0,5
0,4
0,3
0,2
0,1
0
-0,1
200
220
240
260
280
300
[nm]
Day 1 - 0%
Day 1 - 25%
Day 1 - 50%
Day 1 - 60%
Day 1 - 70%
Day 1 - 75%
Day 1 - 80%
Day 1 - 100%

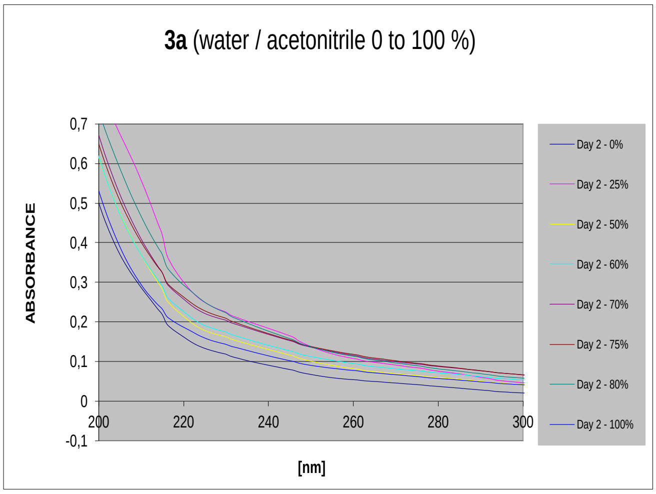
3a (water / acetonitrile 0 to 100 %)
ABSORBANCE
0,7
0,6
0,5
0,4
0,3
0,2
0,1
0
-0,1
200
220
240
260
280
300
[nm]
Day 2 - 0%
Day 2 - 25%
Day 2 - 50%
Day 2 - 60%
Day 2 - 70%
Day 2 - 75%
Day 2 - 80%
Day 2 - 100%

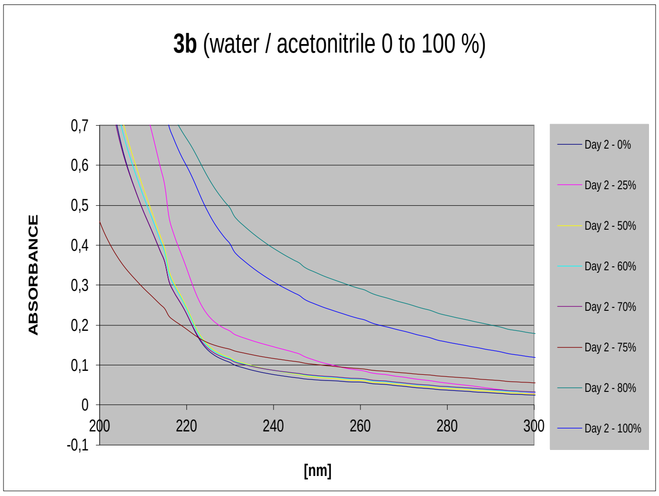
3b (water / acetonitrile 0 to 100 %)
ABSORBANCE
0,7
0,6
0,5
0,4
0,3
0,2
0,1
0
-0,1
200
220
240
260
280
300
[nm]
Day 2 - 0%
Day 2 - 25%
Day 2 - 50%
Day 2 - 60%
Day 2 - 70%
Day 2 - 75%
Day 2 - 80%
Day 2 - 100%

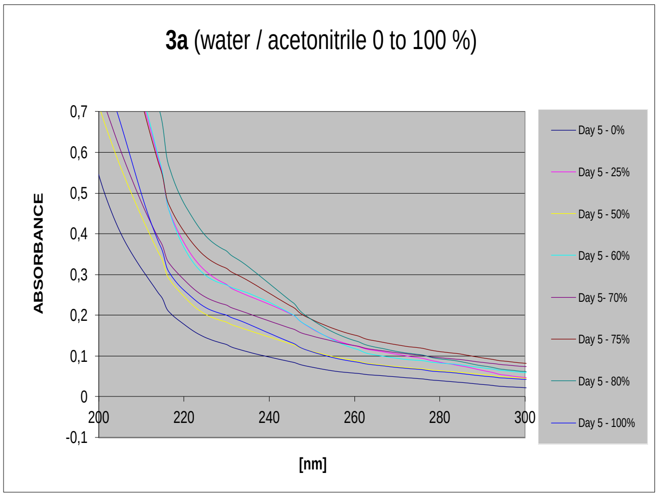
3a (water / acetonitrile 0 to 100 %)
ABSORBANCE
0,7
0,6
0,5
0,4
0,3
0,2
0,1
0
-0,1
200
220
240
260
280
300
[nm]
Day 5 - 0%
Day 5 - 25%
Day 5 - 50%
Day 5 - 60%
Day 5- 70%
Day 5 - 75%
Day 5 - 80%
Day 5 - 100%

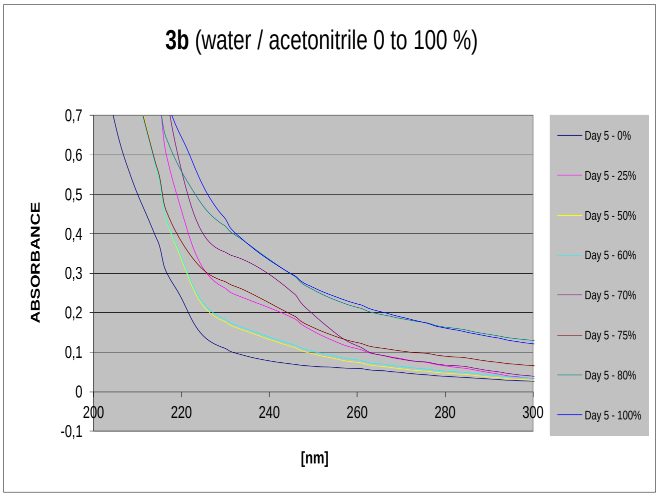
3b (water / acetonitrile 0 to 100 %)
ABSORBANCE
0,7
0,6
0,5
0,4
0,3
0,2
0,1
0
-0,1
200
220
240
260
280
300
[nm]
Day 5 - 0%
Day 5 - 25%
Day 5 - 50%
Day 5 - 60%
Day 5 - 70%
Day 5 - 75%
Day 5 - 80%
Day 5 - 100%

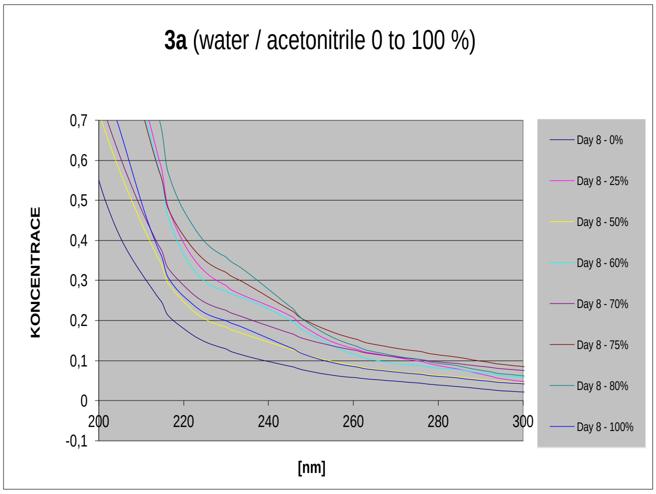
3a (water / acetonitrile 0 to 100 %)
KONCENTRACE
0,7
0,6
0,5
0,4
0,3
0,2
0,1
0
-0,1
200
220
240
260
280
300
[nm]
Day 8 - 0%
Day 8 - 25%
Day 8 - 50%
Day 8 - 60%
Day 8 - 70%
Day 8 - 75%
Day 8 - 80%
Day 8 - 100%

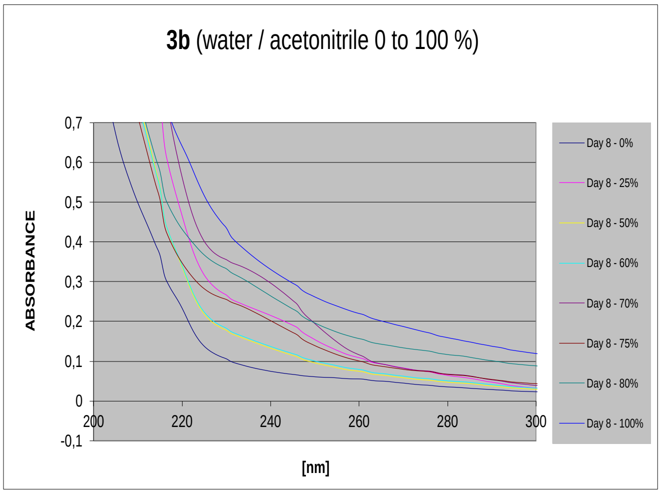
3b (water / acetonitrile 0 to 100 %)
ABSORBANCE
0,7
0,6
0,5
0,4
0,3
0,2
0,1
0
-0,1
200
220
240
260
280
300
[nm]
Day 8 - 0%
Day 8 - 25%
Day 8 - 50%
Day 8 - 60%
Day 8 - 70%
Day 8 - 75%
Day 8 - 80%
Day 8 - 100%
