# Supplementary material for: Stigmasterol-Based Novel Low Molecular Weight/Mass Organic Gelators
Source: Molecules. 2011 Nov 8;16(11):9357–67. doi: 10.3390/molecules16119357 (PMC6264699; doi:10.3390/molecules16119357)
Supplement: Supplementary File 2 [file molecules-16-09357-s002.pdf]

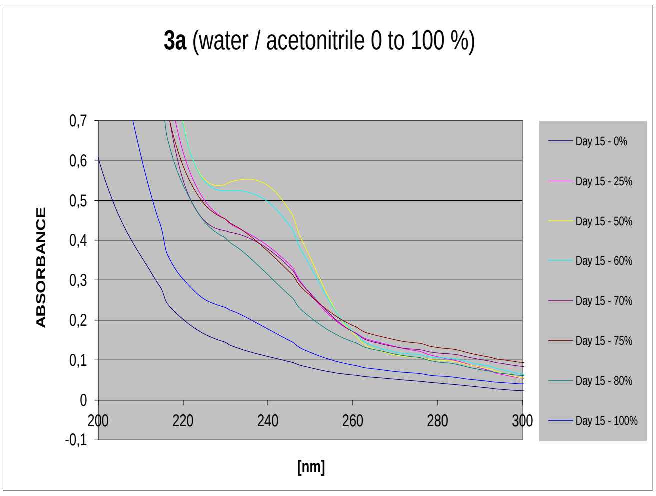
3a (water / acetonitrile 0 to 100 %)
ABSORBANCE
[nm]
Day 15 - 0%
Day 15 - 25%
Day 15 - 50%
Day 15 - 60%
Day 15 - 70%
Day 15 - 75%
Day 15 - 80%
Day 15 - 100%

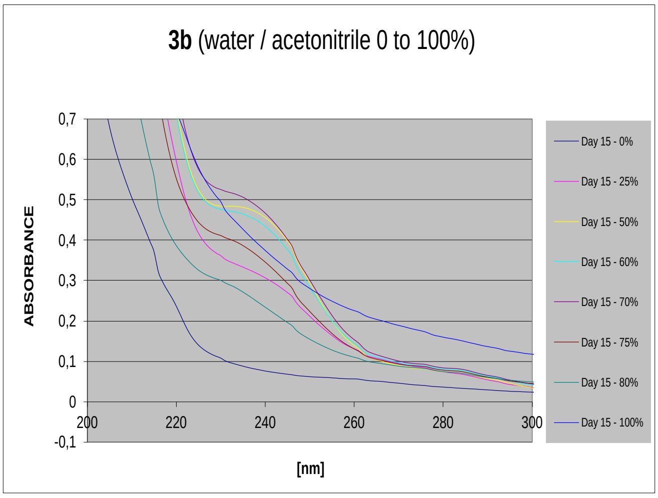
3b (water / acetonitrile 0 to 100%)
ABSORBANCE
[nm]
Day 15 - 0%
Day 15 - 25%
Day 15 - 50%
Day 15 - 60%
Day 15 - 70%
Day 15 - 75%
Day 15 - 80%
Day 15 - 100%

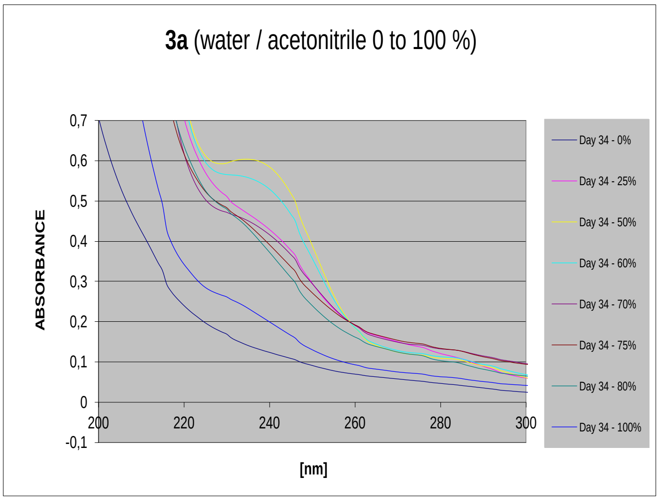
3a (water / acetonitrile 0 to 100 %)
ABSORBANCE
[nm]
Day 34 - 0%
Day 34 - 25%
Day 34 - 50%
Day 34 - 60%
Day 34 - 70%
Day 34 - 75%
Day 34 - 80%
Day 34 - 100%

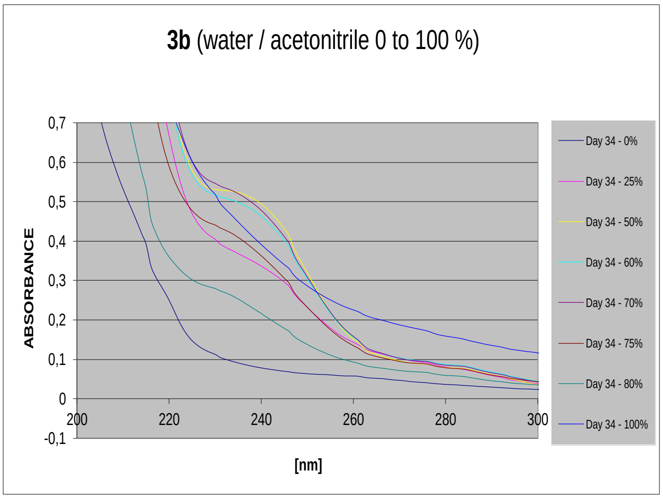
3b (water / acetonitrile 0 to 100 %)
ABSORBANCE
[nm]
Day 34 - 0%
Day 34 - 25%
Day 34 - 50%
Day 34 - 60%
Day 34 - 70%
Day 34 - 75%
Day 34 - 80%
Day 34 - 100%

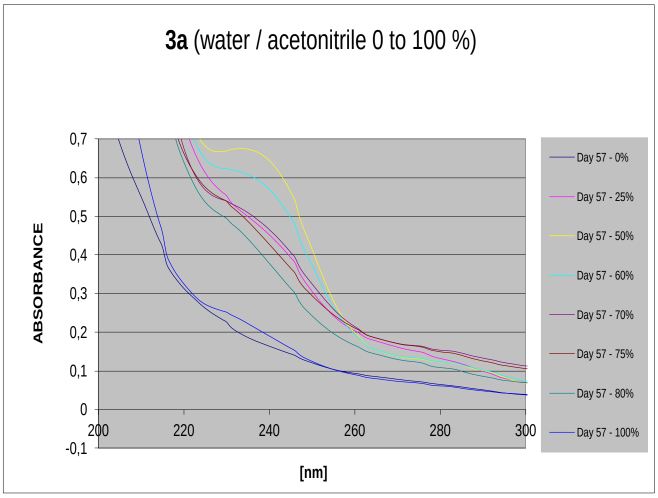
3a (water / acetonitrile 0 to 100 %)
ABSORBANCE
[nm]
Day 57 - 0%
Day 57 - 25%
Day 57 - 50%
Day 57 - 60%
Day 57 - 70%
Day 57 - 75%
Day 57 - 80%
Day 57 - 100%

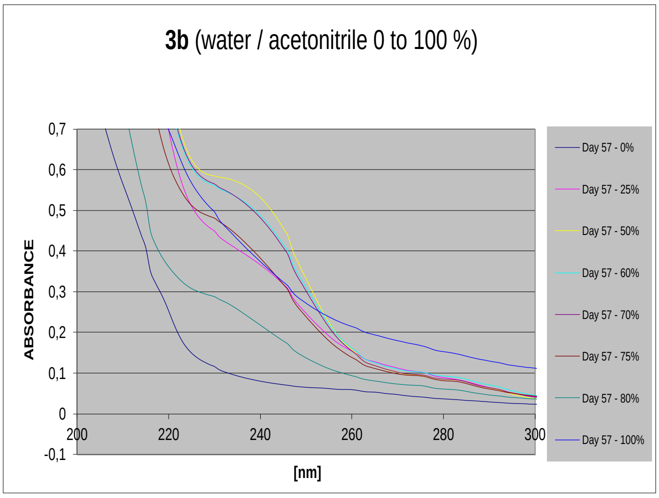
3b (water / acetonitrile 0 to 100 %)
ABSORBANCE
[nm]
Day 57 - 0%
Day 57 - 25%
Day 57 - 50%
Day 57 - 60%
Day 57 - 70%
Day 57 - 75%
Day 57 - 80%
Day 57 - 100%
